# Diastereoselective radical addition to γ-alkyl-α-methylene-γ-butyrolactams and the synthesis of a chiral pyroglutamic acid derivative

**DOI:** 10.3762/bjoc.9.161

**Published:** 2013-07-17

**Authors:** Tomoko Yajima, Eriko Yoshida, Masako Hamano

**Affiliations:** 1 Department of Chemistry, Ochanomizu University, Otsuka, Bunkyo-ku, Tokyo, 112-8610, Japan

**Keywords:** chelation controlled reaction, diastereoselective reaction, free radical, lactams, pyroglutamic acid derivative, radical alkylation

## Abstract

The *cis*- and *trans*-stereoselective radical additions to α-methylene-γ-alkyl- γ-lactams were investigated and the scope and limitation of the reaction were also revealed. This stereoselective radical reaction was used for synthesis of chiral pyroglutamic acid derivatives starting from a commercially available chiral amino acid.

## Introduction

γ-Lactams exist in many natural products and biologically active compounds and are one of the most important classes of compounds for drug discovery [1–3]. Substituted γ-lactams, in particular, have potential application in drug synthesis, but the development of stereoselective synthesis of chiral γ-lactams remains a challenge [4–5]. Developing effective and simple synthetic methods is important so that the drug candidates can be screened. A stereoselective addition to a γ-lactam skeleton provides a direct and efficient method for synthesizing various γ-lactam derivatives. However, the most commonly used methods for synthesizing chiral γ-lactams are based on the cyclization or cycloaddition of *N*-containing precursors, which are synthesized stereoselectively, and there are limited studies on the stereoselective additions to γ-lactam skeletons [6–8] and no reports on radical addition.

**Scheme 1:**
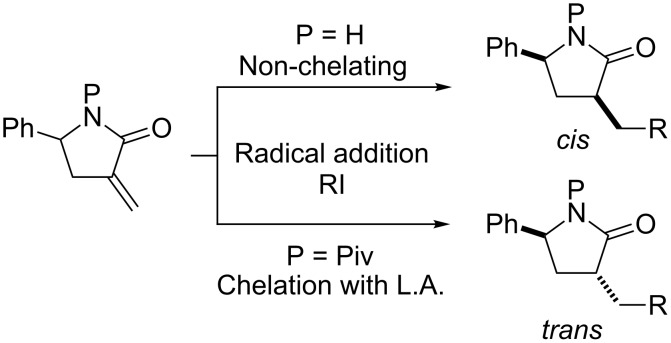
Radical addition to α-methylene-γ-phenyl-γ-butyrolactams.

We have already investigated diastereoselective alkyl radical additions to α-methylene-γ-phenyl- γ-lactam and reported that the *N*-unsubstituted lactam yields *cis*-α,γ-disubstituted lactams using (Me_3_Si)_3_SiH under UV irradiation, whereas the reactions of *N*-pivaloyllactams with Et_3_B and Bu_3_SnH in the presence of Yb(OTf)_3_ yields *trans*-α,γ-disubstituted lactams, both reactions involving various alkyl radicals (Scheme 1) [9]. Although this method allows the stereoselective introduction of various substituents into γ-lactams, only γ-phenyl-γ-lactam was used as a substrate. Therefore, we were interested in whether our reaction conditions would be suitable for γ-alkyl substrates and would allow the efficient synthesis of chiral *N*-containing compounds.

Here, we report *cis*- and *trans*-stereoselective radical additions to α-methylene-γ-alkyl- γ-lactams and the synthesis of chiral pyroglutamic acid derivatives using our reaction, starting from a commercially available chiral amino acid.

## Results and Discussion

First, *cis*-selective isopropyl radical additions to α-methylene-γ-alkyl-γ-lactams were investigated (Table 1). The lactams **1a**–**1d** were synthesized following published procedures [10]. The conditions used in our previous study [9] were used as the starting point, and the reactions of **1a**–**1d** (1 equiv) with isopropyl iodide (3 equiv) in CH_2_Cl_2_ by using AIBN (0.2 equiv) as a radical initiator and (Me_3_Si)_3_SiH (TTMSS) (2 equiv) as a H-donor were performed at room temperature under UV irradiation. The reactions of **1a** and **1b** yielded strong *cis*-diastereoselectivities, but the reactions of **1c** and **1d** were less diastereoselective.

**Table 1:** Radical addition to **1** under non-chelating conditions.

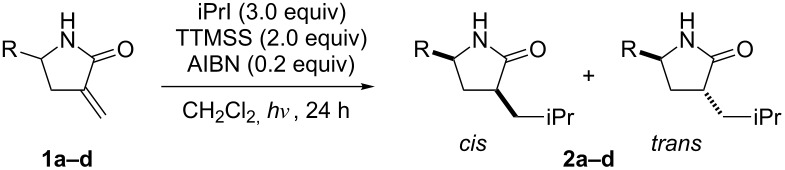					

entry	**1**	R	**2**	yield (%)	*cis*/*trans*

1	**1a**	iPr	**2a**	82	91:9
2	**1b**	*c*-Hex	**2b**	55	92:8
3	**1c**	iBu	**2c**	52	80:20
4	**1d**	PhCH_2_CH_2_	**2d**	49	84:16

Next, isopropyl radical additions to *N*-pivaloyl substrates **3a**–**3d** in the presence of a Lewis acid were investigated (Table 2). The reactions of **3a**–**3d** (1 equiv) with isopropyl iodide (3 equiv) in CH_2_Cl_2_ in the presence of Et_3_B (1 equiv), Bu_3_SnH (2 equiv), and Yb(OTf)_3_ (0.5 equiv) were performed at −78 °C. The reactions of **3a**, **3c,** and **3d** were almost nondiastereoselective, while that of **3b** was *cis*-selective. The selectivity was almost the same as when the reaction was performed without a Lewis acid (Table 2, entry 3). Using an excess of Yb(OTf)_3_ did not affect the diastereoselectivity (Table 2, entry 2).

**Table 2:** Radical addition to **3** under chelating conditions.

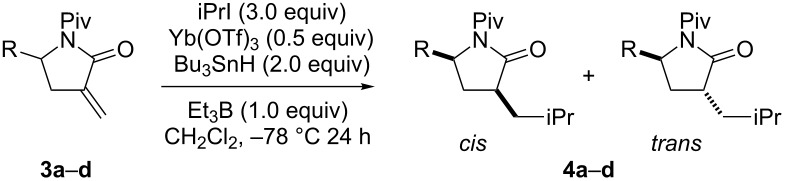					

entry	**3**	R	**4**	yield (%)	*cis*/*trans*

1	**3a**	iPr	**4a**	90	45:55
2	**3b**	*c*-Hex	**4b**	42	75:25
3^a^	**3b**	*c*-Hex	**4b**	62	88:12
4^b^	**3b**	*c*-Hex	**4b**	46	80:20
5	**3c**	iBu	**4c**	85	36:64
6	**3d**	PhCH_2_CH_2_	**4d**	71	50:50

^a^Without Yb(OTf)_3_. ^b^1.0 equiv of Yb(OTf)_3_ was used.

We then changed the protecting group of the lactam nitrogen from the pivaloyl to the acetyl group and investigated the reaction in the presence of a Lewis acid (Table 3). The reactions of **5c** and **5d** yielded strong *trans*-selectivities, but *cis*-selectivities were observed in the reactions of **5a** and **5b**. The use of MgBr_2_–OEt_2_ instead of Yb(OTf)_3_ enhanced the yield but did not affect the diastereoselectivity (Table 3, entry 2).

**Table 3:** Radical addition to **5** under chelating conditions.

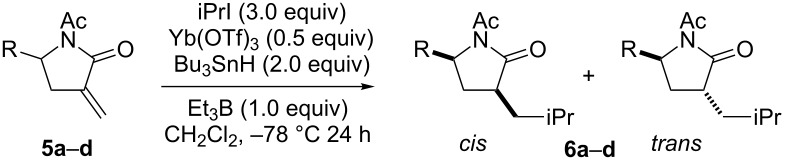					

entry	**5**	R	**6**	yield (%)	*cis*/*trans*

1	**5a**	iPr	**6a**	37	78:22
2^a^	**5a**	iPr	**6a**	84	66:39
3	**5b**	*c*-Hex	**6b**	51	62:38
4	**5c**	iBu	**6c**	97	20:80
5	**5d**	PhCH_2_CH_2_	**6d**	92	25:75

^a^3.0 equiv of MgBr_2_–OEt_2_ was used instead of Yb(OTf)_3_.

**Figure 1:**
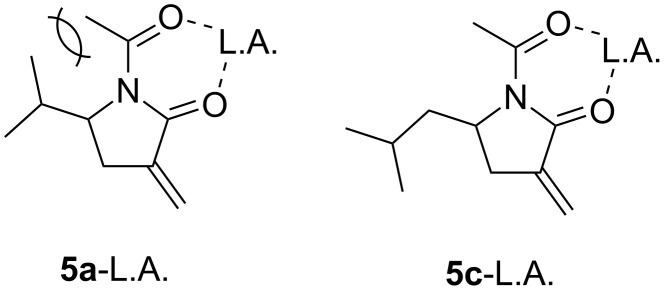
Chelation of **5a** and **5d**.

The direction of hydrogen transfer from the H-donor (*n*-Bu_3_SnH or TTMSS) to the intermediate radical determined the stereochemistry of the product. In the case of the reaction of unprotected lactam by using a bulky H-donor (Table 1), hydrogen transferred from the opposite side of the γ-alkyl group and the *cis*-product was obtained. The less hindered primary isobutyl or phenethyl substrates (**1c** or **1d**) yielded poorer stereoselectivities than did **1a** or **1b**. *Trans*-selectivity was expected for the amide type *N*-protecting group reaction because of the coordinating carbonyl oxygens. The carbonyl oxygen of lactam and that of the *N*-protecting group (pivaloyl or acetyl) bidentately coordinate to the Lewis acid to form a six-membered chelate. The Lewis acid coordinates from the opposite side of the γ-alkyl group, and hydrogen transfer occurred from the same face as the γ-alkyl substituent to give *trans*-selectivity. However, strong *trans*-selectivities were not observed for substrates with pivaloyl groups, and it appears that steric hindrance between the pivaloyl group and the lactam γ-alkyl disturbs chelation. Using the acetyl group, which is less sterically hindered than the pivaloyl group, improved the chelate formation of γ-isobutyl or γ-phenethyl substrates and the reactions of **5c** and **5d** were *trans*-selective. In contrast, relatively bulky isopropyl or cyclohexyl groups could not form the six-membered chelate because of the steric repulsion between the acetyl and tertiary alkyl group (Figure 1).

**Scheme 2:**
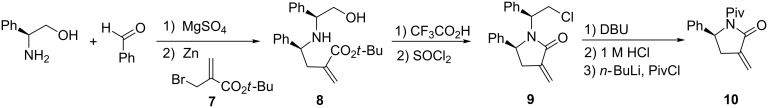
Synthesis of chiral substrate **10**.

Finally, we attempted to synthesize the chiral pyroglutamic acid derivatives starting from a commercially available chiral amino acid. The reaction of benzaldehyde and (*S*)-phenylglycinol in the presence of MgSO_4_ (used as a dehydrating reagent) gave a chiral imine, and the subsequent Reformatsky reaction with bromide **7** afforded butyl acrylate **8** as a single diastereomer [11–12] (Scheme 2). Hydrolysis with CF_3_CO_2_H and converting the hydroxy group to the chloride yielded the corresponding lactam **9** [13]. The chiral auxiliary was removed by DBU-assisted elimination to the enamine and subsequent hydrolysis [14]. Introducing the pivaloyl group, because *N*-pivaloyl gave high trans-selectivity of γ-phenyl substrate, yielded the chiral radical substrate **10**.

**Scheme 3:**
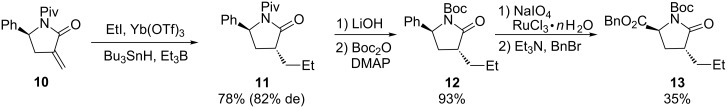
Synthesis of chiral 4-butyl-L-pyroglutamic acid **13**.

The *trans*-selective ethyl radical addition proceeded, yielding **11** with high diastereoselectivity, as we previously reported [9]. The *trans*-isomer was isolated by silica-gel column chromatography. The *N-*pivaloyl group on **11** was converted to the Boc group, because ruthenium oxidation did not proceed on using the pivaloyl-protected substrate **11**. The phenyl group was oxidized to the carboxylic acid by using ruthenium trichloride [15–16], and the benzylation of the carboxyl group yielded the pyroglutamic acid derivative **13** as a single stereoisomer (Scheme 3).

## Conclusion

In this study, we investigated the *cis*- and *trans*-stereoselective radical additions to α-methylene-γ-alkyl- γ-lactams. Strong *cis*-selectivities were observed using various γ-substituents under non-chelating conditions. The reactions of pivaloyl protected substrates in the presence of a Lewis acid were not *trans*-selective, although pivaloyl protected γ-phenyl substrates gave high *trans*-selectivity in our previous report. The poor selectivities were attributed to the steric repulsion between the pivaloyl group and the γ-substituent on the substrate. The reactions using *N*-acetyl substrates instead of pivaloyl substrates yielded better *trans*-selectivities with γ-isobutyl and γ-phenethyl substrates, because acetyl is sufficiently small to allow chelation with the Lewis acid and lactam carbonyl. We used the reaction to synthesize chiral pyroglutamic acid derivatives starting from (*S*)-phenylglycinol.

## Supporting Information

File 1 Experimental procedures and characterization data for compounds **2a**–**d**, **6a**–**d** and **9**–**13**.
