# Supplementary material for: Diastereoselective radical addition to γ-alkyl-α-methylene-γ-butyrolactams and the synthesis of a chiral pyroglutamic acid derivative
Source: Beilstein J Org Chem. 2013 Jul 17;9:1432–6. doi: 10.3762/bjoc.9.161 (PMC3740493; doi:10.3762/bjoc.9.161)
Supplement: File 1 — Experimental procedures and characterization data for compounds 2a–d, 6a–d and 9–13. [file Beilstein_J_Org_Chem-09-1432-s001.pdf]

Supporting Information

for

Diastereoselective radical addition to γ-alkyl-α-methylene-γ-butyrolactams and the synthesis of a chiral pyroglutamic acid derivative

Tomoko Yajima*, Eriko Yoshida and Masako Hamano

Address: Department of Chemistry, Ochanomizu University, Otsuka, Bunkyo-ku, Tokyo, 112-8610, Japan

Email: Tomoko Yajima - yajima.tomoko@ocha.ac.jp

* Corresponding author

Experimental procedures and characterization data for compounds 2a–d, 6a–d and 9–13

General information

^{1}H NMR spectra were recorded on a JEOL AL-400 or EX-400 or Bruker Avance 600 (600 MHz) spectrometer with $CDCl_3$ as the solvent and tetramethylsilane as an internal standard unless otherwise noted. ^{13}C NMR spectra were recorded on the instruments operating at 100.5 MHz or 125.0 MHz with $CDCl_3$ as the solvent and internal standard (δ 77.0). IR spectra were taken on a SHIMADZU FTIR-8700 spectrometer. Mass spectra (EI^+) were obtained on a JEOL JMS-700 mass spectrometer (ESI) or Thermo Exactive (ESI). Precoated Merck Kieselgel 60 F_{254} and Kanto silica gel 60 (spherical neutral) were used for thin-layer chromatography and flash chromatography, respectively. Photoinduced reactions were performed with a high-pressure Hg lamp (Ushio, 450 W).

General procedure for the radical addition to lactam 1a–d (TTMSS / AIBN)

In a Pyrex glass tube were placed olefin (0.15 mmol), isopropyl iodide (0.45 mmol, 3 equiv), AIBN (0.03 mmol, 0.2 equiv), TTMSS (0.3 mmol, 2 equiv) and CH_2Cl_2 (5 mL). After sealing the tube, the mixture was shaken and then irradiated with a Hg lamp at room temperature. The solvent was evaporated in vacuo and the residue was purified by flash chromatography on silica gel to give the product.

3-Isobutyl-5-isopropyl-2-oxopyrrolidine (2a): *cis*/*trans* = 91:9 mixture, White powder; IR (KBr) 3204, 2959, 2874, 1691, 1391, 792, 578 cm^{-1}; ^{1}H NMR (400 MHz) δ 5.62 (1H, bs), 3.30 (1H, m, *trans*-isomer), 3.24 (1H, m, *cis*-isomer), 2.39 (2H, m), 1.79 (1H, m), 1.66 (1H, m), 1.56 (1H, m), 1.26 (2H, m), 0.90 (12H, m); ^{13}C NMR (100 MHz) δ 180.2, 58.7, 40.4, 40.2, 33.6, 33.0, 26.2, 23.6, 21.4, 19.4, 18.0; MS (EI) *m*/*z*: 182 (M^+ − H, 3%), 140 (72), 127 (26), 84 (14), 58 (55), 43 (100); HRMS: calcd for $C_{11}H_{21}NO$, 183.1589; found, 183.1606.

5-Cyclohexyl-3-isobutyl-2-oxopyrrolidine (2b): *cis*/*trans* = 92:8 mixture, White powder; IR (KBr) 3202, 2933, 2854, 1679, 1454, 797, 576 cm^{-1}; ^{1}H NMR (400 MHz) δ 5.61 (1H, bs), 3.30 (1H, m, *trans*-isomer), 3.24 (1H, m, *cis*-isomer), 2.36 (2H, m), 1.82-1.15 (15H, m), 0.94 (3H, d, J = 6.3 Hz), 0.89 (3H, d, J = 6.3 Hz); ^{13}C NMR (100 MHz) δ 180.1, 57.7, 43.5, 40.4, 40.0, 33.2, 30.0, 28.5, 26.3, 26.2, 25.8, 23.6, 21.3; MS (EI) *m*/*z*: 208 (M^+ − NH, 2%), 167 (38), 140 (96), 127 (11), 85 (16), 58 (100); HRMS: calcd for $C_{14}H_{25}NO$, 223.1941; found, 223.1939.

3,5-Diisobutyl-2-oxopyrrolidine (2c): *cis*/*trans* = 80:20 mixture, White powder; IR (KBr) 3207, 2951, 2887, 1691, 1390, 788, 594 cm^{-1}; ^{1}H NMR (400 MHz) δ 6.25 (1H, bs), 3.68 (1H, m, *trans*-isomer), 3.61 (1H, m, *cis*-isomer), 2.41 (2H, m), 1.79-1.27 (7H, m), 0.96-0.92 (9H, m), 0.89 (3H, d, *J* = 6.3 Hz); ^{13}C NMR (100 MHz) δ 180.2, 50.8, 46.2, 40.4, 40.1, 33.2, 36.0, 26.1, 25.4, 23.5, 22.7, 22.6, 21.3; HRMS (ESI^{+}) *m*/*z*: calcd for $C_{12}H_{23}NONa$ $[M + Na]^{+}$ 220.1672; found, 220.1975

3-Isobutyl-2-oxo-5-(2-phenylethyl)pyrrolidine (2d): *cis*/*trans* = 84:16 mixture, White powder; IR (KBr) 3203, 2955, 2852, 1693, 1472, 1455, 1386, 1304, 791, 765, 738, 702, 568 cm^{-1}; ^{1}H NMR (400 MHz) δ 7.31-7.17 (5H, m), 7.03 (1H, bs, *trans*-isomer), 6.93 (1H, bs, *cis*-isomer), 3.55 (1H, m), 2.67 (1H, t, *J* = 8.0 Hz), 2.44 (2H, m), 1.80 (3H, m), 1.66 (1H, m), 1.26 (2H, m), 0.93 (3H, d, *J* = 6.4 Hz), 0.89 (3H, d, *J* = 6.4 Hz); ^{13}C NMR (100 MHz) δ 180.0, 140.9, 128.5 (2C), 128.2 (2C), 126.1, 52.2, 40.5, 40.1, 38.6, 35.5, 32.5, 26.2, 23.5, 21.4; MS (EI) *m*/*z*: 230 (M^{+} − NH, 5%), 202 (M^{+} − NH, CO, 13), 189 (100), 140 (59), 91 (36), 85 (71),43 (34); HRMS: calcd for $C_{16}H_{23}ON$, 245.1810; found, 245.1795.

General procedure for the radical addition to lactam 5a–d (Bu_3SnH / Et_3B / L. A.)

To a solution of olefin (0.15 mmol) in dry CH_2Cl_2 (1.5 mL) was added $Yb(OTf)_3$ (0.45 mmol, 3 equiv), and the mixture was stirred at room temperature for 10 min. To the suspension cooled to −78 °C were added isopropyl iodide (0.45 mmol, 3 equiv), *n*-Bu_3SnH (0.30 mmol, 2 equiv) and Et_3B (1.06 mol/L in hexane; 0.15 mmol, 1 equiv). The mixture was stirred at −78 °C for 3 h. KF and water were added and the reaction mixture was stirred at room temperature for 3 h. After filtration, the solvent was evaporated in vacuo. The residue was purified by flash chromatography on silica gel to give the product.

1-Acetyl-3-isobutyl-5-isopropyl-2-oxopyrrolidine (6a): *cis*/*trans* = 78:22 mixture, Colorless oil; IR (neat) 2960, 1734, 1700, 1468, 1372, 1270, 1180, 1111, 842, 610 cm^{-1}; ^{1}H NMR (400 MHz) δ *cis*-isomer: 4.15 (1H, m), 2.70 (1H, m), 2.52 (1H, m), 2.50 (1H, m), 2.13 (1H, m), 1.76 (2H, m), 1.32 (2H, m), 0.97 (3H, d, *J* = 6.4 Hz), 0.91 (3H, d, *J* = 6.4 Hz), 0.88 (3H, d, *J* = 6.8 Hz), 0.66 (3H, d, *J* = 6.8 Hz), *trans*-isomer: 4.23 (1H, ddd, *J* = 9.2, 4.4, 1.6 Hz), 2.66 (1H, m), 2.51 (1H, m), 2.26 (1H, m), 2.07 (1H, m), 1.80-1.52 (2H, m), 1.39 (1H, m), 1.24 (1H, m), 0.95 (3H, d, *J* = 6.4 Hz), 0.94 (3H, d, *J* = 6.4 Hz), 0.91 (3H, d, *J* = 6.4 Hz), 0.83 (3H, d, *J* = 6.4 Hz); ^{13}C NMR (100 MHz) 178.9, 171.9, 59.7, 40.6, 40.1, 27.0, 26.1, 26.0, 23.4,

23.4, 23.3, 21.4, 18.3, 14.2; MS (EI) *m*/*z*: 225 (M^+, 24%), 182 (39), 169 (15), 140 (100); HRMS: calcd for $C_{13}H_{23}NO_2$, 225.1723; found, 225.1726.

1-Acetyl-5-cyclohexyl-3-isobutyl-2-oxopyrrolidine (6b): *cis*/*trans* = 62:38 mixture, Colorless oil; IR (neat) 2930, 1737, 1700, 1451, 1370, 1267, 1173, 1123, 987, 607 cm^{-1}; ^1H NMR (400 MHz) δ *cis*-isomer: 4.12 (1H, m), 2.51 (1H, m), 2.50 (1H, m), 2.32 (1H, m), 2.13 (1H, m), 1.78-1.61 (6H, m), 1.42-1.08 (8H, m), 0.97 (3H, d, *J* = 6.4 Hz), 0.91 (3H, d, *J* = 6.4 Hz), *trans*-isomer: 4.21 (1H, m), 2.65 (1H, m), 2.51 (1H, m), 2.13 (1H, m), 1.88-1.55 (8H, m), 1.42-1.07 (7H, m), 0.95 (3H, d, *J* = 6.4 Hz), 0.93 (3H, d, *J* = 6.4 Hz); ^{13}C NMR (100 MHz) δ 178.9, 171.9, 59.4, 40.8, 40.2, 37.7, 28.9, 26.6, 26.3, 26.1, 26.0, 25.7, 24.8, 24.7, 23.4, 21.4; MS (EI) *m*/*z*: 265 (M^+, 4%), 209 (22), 140 (100), 55 (18) , 43 (25); HRMS: calcd for $C_{16}H_{27}NO_2$, 265.2124; found, 265.2083.

1-Acetyl-3,5-diisobutyl-2-oxopyrrolidine (6c): *cis*/*trans* = 20:80 mixture, Colorless oil; IR (neat) 2959, 2873, 1739, 1699, 1469, 1373, 1353, 1279, 1192, 1135, 1107, 905, 720, 625, 604 cm^{-1}; ^1H NMR (400 MHz) δ 4.32 (1H, m), 2.69 (1H, m), 2.57-2.36 (1H, m), 2.48 (3H, s), 2.03 (1H, m), 1.82-1.54 (4H, m), 1.42-1.25 (1H, m), 0.99-0.90 (12H, m); ^{13}C NMR (100 MHz) δ 177.7, 170.6, 53.3, 41.5, 40.2, 39.8, 30.2, 25.8, 25.7, 25.5, 23.7, 23.3 (2C), 21.5; MS (EI) *m*/*z*: 239 (M^+, 8%), 196 (29), 183 (61), 182 (24), 168 (59), 142 (27), 140 (100), 127 (98), 85(32); HRMS: calcd for $C_{14}H_{25}NO_2$, 239.1835; found, 239.1860.

1-Acetyl-3-isobutyl-2-oxo-5-(2-phenylethyl)pyrrolidine (6d): *cis*/*trans* = 25:75 mixture, Colorless oil; IR (neat) 3028, 2957, 2872, 1739, 1700, 1498, 1455, 1373, 1349, 1279, 1193, 1115, 1040, 907, 749, 700, 624, 604 cm^{-1}; ^1H NMR (400 MHz) δ *trans*-isomer: 7.31-7.17 (5H, m), 4.31 (1H, m), 2.69 (3H, m), 2.48 (3H, s), 2.11 (2H, m), 1.71 (4H, m), 1.25 (1H, m), 0.95 (3H, d, *J* = 6.4 Hz), 0.91 (3H, d, *J* = 6.4 Hz), *cis*-isomer: 7.30-7.17 (5H, m), 4.15 (1H, m), 2.71-2.30 (5H, m), 2.47 (3H, s), 1.76 (3H, m), 1.46 (1H, m), 1.35 (1H, m), 0.97 (3H, d, *J* = 6.4 Hz), 0.91 (3H, d, *J* = 6.4 Hz); ^{13}C NMR (100 MHz) δ 177.7, 170.8, 140.8, 128.4 (2C), 128.1 (2C), 126.0, 54.6, 40.4, 39.9, 34.1, 32.4, 29.9, 25.9, 25.5, 23.4, 21.5; MS (EI) *m*/*z*: 287 (M^+, 87%), 244 (100), 202 (67), 140 (83), 127 (61), 91 (51); HRMS: calcd for $C_{18}H_{25}NO_2$, 287.1820; found, 287.1853.

(*S*)-1-((*S*)-2-Chloro-1-phenylethyl)-3-methylene-2-oxo-5-phenylpyrrolidine (9)

Ester **8** (2.401 g, 6.5 mmol) was dissolved in CH_2Cl_2 (20 mL) and cooled to 0 °C. Trifluoroacetic acid (19.6 mmol) and thionyl chloride (16.4 mmol) were added and the reaction mixture was allowed to warm slowly to rt. Then the mixture was heated under reflux for 16 h, quenched with sat. NH_4Cl (aq) and extracted with CH_2Cl_2. The organic layer was dried over Na_2SO_4, filtered, and concentrated in vacuo. The residue was purified by column chromatography on silica gel to give **9** (1.931 g, 95%). White powder; IR (KBr) 3404, 3033, 1684, 1657, 1427, 1349, 1281, 1135, 771, 701, 643 cm^{-1}; ^{1}H NMR (400 MHz) δ 7.29 (6H, m), 7.11 (4H, m), 6.16 (1H, t, *J* = 2.4 Hz), 5.41 (1H, t, *J* = 2.4 Hz), 5.18 (1H, t, *J* = 7.6 Hz), 4.36 (1H, dd, *J* = 8.4, 4.0 Hz), 4.02 (1H, dd, *J* = 8.4, 8.0 Hz), 3.46 (1H, dd, *J* = 8.0, 7.6 Hz), 3.14 (1H, ddt, *J* = 17.6, 8.4, 2.4 Hz), 2.60 (1H, ddt, *J* = 17.6, 4.0, 2.4 Hz); ^{13}C NMR (150 MHz) 174.3, 141.7, 138.7, 136.8, 128.8 (4C), 128.2 (4C), 128.1, 126.8 (3C), 116.6, 60.0, 59.9, 43.1, 33.1; HRMS (ESI^{+}) *m/z*: $[M + H]^{+}$ calcd for $C_{19}H_{19}ClNO$, 312.1150; found, 312.1144.

(*S*)-1-(2,2-dimethyl-1-oxopropyl)-3-methylene-2-oxo-5-phenylpyrrolidine (10)

Colorless solid; IR (KBr) 2958, 1729, 1686, 1657, 1396, 1365, 1315, 1284, 1166, 1153, 937, 702 cm^{-1}; ^{1}H NMR δ 7.29 (2H, t, *J* = 8.0 Hz), 7.21 (1H, t, *J* = 8.0 Hz), 7.19 (2H, d, *J* = 8.0 Hz), 6.25 (1H t, *J* = 2.4 Hz), 5.47 (1H, t, *J* = 2.4 Hz), 5.36 (1H, dd, *J* = 9.3, 2.9 Hz), 3.19 (1H, ddt, *J* = 17.1, 9.3, 2.4 Hz), 2.64 (1H, ddt, *J* = 17.1, 2.9, 2.4 Hz), 1.35 (9H, s); ^{13}C NMR δ 180.3, 166.2, 142.6, 139.4, 128.7 (2C), 127.5, 125.1 (2C), 120.7, 58.8, 42.0, 34.2, 26.1(3C); MS *m/z*: 257 (M^{+}, 9%), 202 (65), 172 (100), 158 (26), 129 (94), 104 (24), 91 (22), 77 (28), 57 (52); $[\alpha]^{24}_{D}$ −35.7 (*c* 0.03, $CHCl_3$); HRMS: calcd for $C_{16}H_{19}NO_2$, 257.1416; found, 257.1388.

(3*R*,5*S*)-1-(2,2-dimethyl-1-oxopropyl)-2-oxo-5-phenyl-3-propylpyrrolidine (11) *cis*/*trans* = 9:91 mixture, Yellow oil; IR (neat) 2961, 2933, 2874, 1739, 1688, 1456, 1395, 1365, 1267, 1192, 700 cm^{-1}; ^{1}H NMR δ *trans*-isomer: 7.32 (2H, t, *J* = 7.2 Hz), 7.25 (1H, t, *J* = 7.2 Hz), 7.14 (2H, d, *J* = 7.2 Hz), 5.40 (1H, dd, *J* = 7.8, 2.4 Hz), 2.74 (1H, ddd, *J* = 10.4, 8.8, 4.4 Hz), 2.13 (2H, m), 1.87 (1H, m), 1.30-1.45 (3H, m), 1.34 (9H, s), 0.91 (3H, t, *J* = 7.3 Hz); *cis*-isomer: 7.13-7.34 (5H, m), 5.08 (1H, dd, *J* = 9.7, 6.8 Hz), 2.74 (1H, m), 2.60 (2H, m), 1.88 (1H, m), 1.37-1.46 (3H, m), 1.25 (9H, s), 0.92 (3H, t, *J* = 6.8 Hz); ^{13}C NMR δ 179.2, 175.9, 141.8, 128.6 (2C), 127.1, 124.7 (2C), 59.8, 42.1, 41.7, 34.1, 32.7, 26.0 (3C), 20.1, 14.0; MS *m/z*: 287 (M^{+}, 2%), 245 (11), 202 (45), 132 (45), 104 (38), 77 (46), 57 (100), 41 (67), 29 (27); $[\alpha]^{24}_{D}$ −23.8 (*c* 0.07, $CHCl_3$); HRMS: calcd for $C_{18}H_{25}NO_2$, 287.1885; found, 287.1869.

(3*R*,5*S*)-*tert*-butyl 2-oxo-5-phenyl-3-propylpyrrolidine-1-carboxylate (12) IR (neat) 2958, 2362, 1783, 1747, 1716, 1367, 1304, 1152, 948, 852, 700, 668 cm^{-1}; ^{1}H NMR (400 MHz, $CDCl_3$) δ 7.35 (2H, m), 7.29 (1H, m), 7.19 (2H, m), 5.14 (1H, dd, *J* = 8.4, 2.4 Hz), 2.66 (1H, m), 2.12 (1H, m), 1.92 (1H, m), 1.38 (1H, m), 1.31 (9H, s), 0.91 (3H, t, *J* = 7.2 Hz); ^{13}C NMR (150 MHz, $CDCl_3$) δ176.6, 149.8, 128.8, 128.7, 127.4, 124.9, 82.8, 59.4, 41.0, 34.2, 32.7, 29.2, 27.8, 27.6, 27.4, 20.3, 13.9; HRMS (ESI^{+}) *m*/*z*: calcd for $C_{18}H_{25}NO_3Na$ [M + Na]$^{+}$ 326.1727; found, 326. 1721

(2*S*,4*R*)-2-benzyl 1-*tert*-butyl 5-oxo-4-propylpyrrolidine-1,2-dicarboxylate (13) Sodium periodate (6.84 mmol) and a catalytic amount of ruthenium(III) chloride *n*H_2O (0.07 mmol) were successively added to a solution of lactam **12** (0.36 mmol) in acetonitrile (3 mL), tetrachloromethane (3 mL) and water (4.5 mL). The solution was heated at 40 °C for 2 hours, then, cooled to room temperature and filtered through a pad of Celite. The residue was concentrated in vacuo to give the crude mixture. To a solution of the crude mixture in THF (9 mL), was added triethylamine (1.44 mmol) and benzyl bromide (0.47 mmol) at 0 °C. After the mixture had been stirred for 12 h at room temperature, the solvent was evaporated in vacuo. The residue was dissolved in 30 mL of CH_2Cl_2, washed with 1 N HCl, sat. $NaHCO_3$ aq, and H_2O, and then dried over Na_2SO_4. The mixture was filtered and concentrated in vacuo. The residue was purified by column chromatography on silica gel to give **13** (45.4 mg, 35%). IR (neat) 2978.5, 2931.9, 1790.9, 1740.0, 1733.6, 1318.2, 1250.5, 1124.1 cm^{-1}; ^{1}H NMR (400 MHz, $CDCl_3$) δ 7.36 (5H, s), 5.23 (1H, d, *J* = 12.0 Hz), 5.19 (1H, d, *J* = 12.0 Hz), 4.59 (1H, dd, *J* = 9.6, 1.6 Hz), 2.53 (1H, m), 2.20 (1H, ddd, *J* = 13.6, 8.8, 1.6 Hz), 1.89 (2H, m), 1.41 (9H, s), 1.32 (4H, m), 0.91 (3H, t, *J* = 7.2 Hz); ^{13}C NMR (150 MHz, $CDCl_3$) δ 175.7, 171.4, 170.7, 149.5, 135.0, 128.7, 128.6, 83.7, 67.3, 57.4, 37.5, 29.6, 27.8, 27.4, 20.3, 13.9; $[\alpha]^{27}_{D}$ −49.5 (*c* 0.04, $CHCl_3$); HRMS (ESI^{+}) *m*/*z*: calcd for $C_{20}H_{27}NO_5Na$ [M + Na]$^{+}$ 384.1781; found, 384.1776.
